# Highlights from the 55th Bürgenstock Conference on Stereochemistry 2022

**DOI:** 10.1039/d2sc90155b

**Published:** 2022-08-18

**Authors:** Alexis Archambeau, Martina Delbianco

**Affiliations:** Laboratoire de Synthèse Organique, UMR 7652, Ecole Polytechnique, ENSTA Paris, CNRS, Institut Polytechnique de Paris 91128 Palaiseau Cedex France alexis.archambeau@polytechnique.edu; Department of Biomolecular Systems, Max Planck Institute of Colloids and Interfaces Am Mühlenberg 1 14476 Potsdam Germany martina.delbianco@mpikg.mpg.de

## Abstract

In May 2022, the 55th Bürgenstock Conference on Stereochemistry happened in person once again. This summary provides insight into the scientific themes discussed during the most recent meeting of this historic and multi-disciplinary conference.
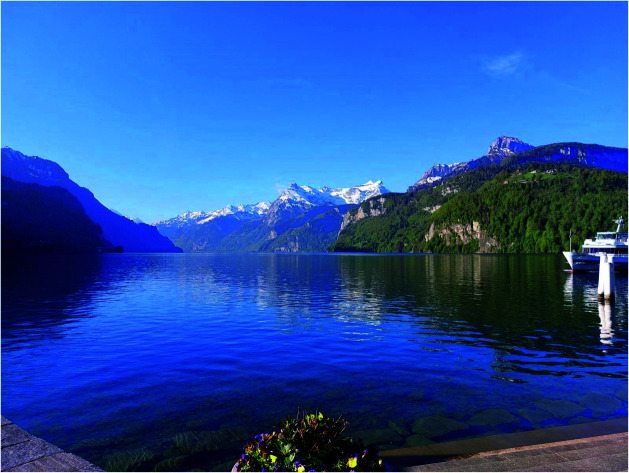

## Introduction

After the cancellation of the conference in 2020 and the online version in 2021, the 55th Bürgenstock Conference on Stereochemistry 2022 has finally happened in person. Young scientists and established professors came together for a five day scientific discussion in the familiar setting of the Seehotel Waldstätterhof in Brunnen, Switzerland. Historically, this conference had strong focus on stereochemistry, but over the years, it has become a multi-disciplinary conference where frontier science is discussed.

The one-hour lectures cover many areas of chemistry and relevant highlights from neighboring disciplines, accommodating long discussions afterwards. Young scientists get the chance to present their work in the form of short presentations or posters. In line with tradition, the program and the names of the invited speakers remain secret until the very first day of the conference.

On May 1st, the organizing committee, headed by the 2022 President Prof. Janine Cossy (ESPCI Paris), welcomed the 120 participants with drinks on a terrace with a stunning view of the Swiss Alps. The sun was shining thanks to the vice-president Prof. Alois Fürstner (Max-Planck-Institut für Kohlenforschung), traditionally in charge of the weather for the entire week. The crowd was a little bigger than usual since it included the 2020/21 JSP fellows that did not have the chance to participate last year, due to Covid-19 restrictions. For many participants, this was the first in person conference after two years of online meetings. The feeling was still a bit surreal, but a few drinks helped break the ice. During the welcome event, the first hints of the program were revealed and Prof. Cossy introduced the guest of honor, the Nobel laureate Prof. Jean-Marie Lehn (University of Strasbourg).

The conference included 13 plenary lectures, 8 short presentations, and 55 posters. In addition, coffee breaks, meals, three evening get-togethers, and a free afternoon were planned, to continue the scientific discussion and promote networking. A quick look at the program suggested that the main theme of this year was the molecular understanding of chemical processes. The plenary lecture covered very broad aspects of chemistry, including heterogeneous catalysis, synthetic methodology, biological and enzymatic processes, and polymerization reactions.

In the next few paragraphs, we give an overview of the science discussed during the meeting. For this report, we decided to borrow an idea expressed by one of the plenary speakers and use it as a tool for our report: Prof. Brian Stoltz (California Institute of Technology) suggested that there are three main reasons that make us interested in organic chemistry – structure, reactivity, function. Thus, we organized all the speakers in these three categories. We want to clarify that the classification is based on our personal opinion and we are aware that most groups likely fit in more than one category. We hope that no one will get offended if they find themselves “misplaced”.

## Structure

Prof. Brian Stoltz was the first speaker to answer this question, revealing that his main interest is structure. He is fascinated by the structure of natural products and his research focuses on the developments of new concepts and methods in organic synthesis to synthesize these complex molecules. His lecture described his efforts toward the construction of challenging all-carbon quaternary centers. Using chiral Pd-catalysts, his group achieved enantioselective formation of quaternary centers by decarboxylative asymmetric allylic alkylation.^1^ This method could be applied to the synthesis of important heterocycles such as lactams and piperazines. Always driven by the passion to generate challenging structures, his group is currently exploring new Ni-catalyzed reactions and Ir-catalyzed asymmetric construction of vicinal quaternary centers.

The structures of some natural products also inspired Prof. David Procter (University of Manchester). His group uses SmI_2_ as a tool for complex organic transformations, for example for the formation of C–C bonds from carboxylic acids, esters, and amides and cyclization cascades by radical relays.^2^ SmI_2_ allows for single electron transfer and its reactivity can be modified with simple additives, such as water. Such a tool permitted the total synthesis of complex molecules to confirm the structure of extracted natural product phaeocaulisin A and allowed access to bicyclo[2.1.1]hexane bioisosteres *via* a catalytic alkene insertion approach.

**Fig. 1:**
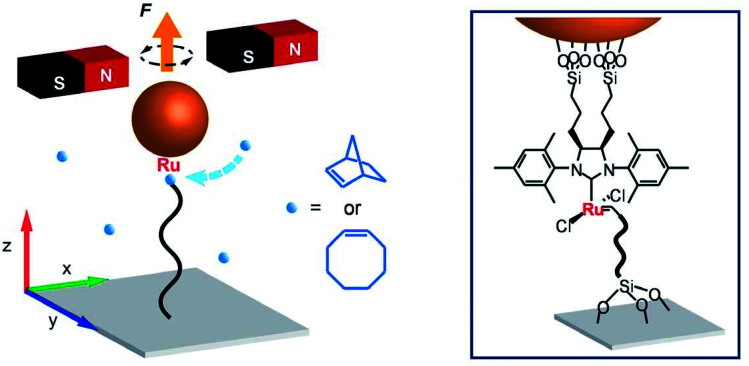
Magnetic tweezers: Prof. Chen's strategy to monitor the ROMP of norbornene or cyclooctene. Reproduced from ref. 3 with permission from the American Association for the Advancement of Science, © 2017.

The interest in the structure of polymers drives the research in Prof. Peng Chen's group (Cornell University). During polymerization, the conformation of growing polymers affects the reactivity. By attaching one end of a growing polymer to a magnetic bead, it was possible to monitor the ring-opening metathesis polymerization of norbornene catalyzed by a Grubbs' catalyst in real time.^3^ This method, called magnetic tweezers, revealed that polymerization follows steps of fast elongation and pauses, due to conformational entanglements of the growing chain (Fig. 1). An alternative method to follow polymer growth is single molecule fluorescence. With this approach, single biopolymer growth can be visualized, allowing for sequence–property correlations.

The previous examples show how the structure of a target could be the driving force to develop new synthetic methodologies and analytical techniques. Prof. Javier Pèrez-Ramírez (ETH Zurich) stressed the importance of synthetic, analytical, and macroscopic tools to understand the structure of heterogeneous catalysts. His team studies the atomic architecture of supported metals to optimize their catalytic performance. They generated a platform of carbon-supported metal catalysts with different nanostructures, from single atoms to nanoparticles, to explore the metal-dependent speciation and host effects in acetylene hydrochlorination.^4^ The importance of a catalyst atomic architecture was also demonstrated with the development of single-atom and bi-metallic heterogeneous catalysts for cross-coupling.

## Reactivity

The interest in structure is directly connected to the interest in reactivity. Indeed, to access complex chemical structures, new reactive pathways have to be developed. Similarly, establishing structure–property correlations is fundamental to develop more efficient reaction methods or catalysts.

Prof. Shū Kobayashi (The University of Tokyo) has inspired everyone by presenting how to improve the sustainability of traditional methods in organic chemistry. He demonstrated that Lewis acid-catalyzed reactions can be more efficient when run in water.^5^ One trick is to integrate a catalyst and its ligand into a carbon nanotube acting as a hydrophobic pocket. Prof. Kobayashi also showcased his expertise in continuous-flow synthesis for the multi-steps preparation of bioactive compounds. A real tour de force!^6^

The reactivity of Grignard reagents is at the core of Prof. Syuzanna Harutyunyan's research interests (University of Groningen). While everyone has heard about these magnesium species during their first organic chemistry classes, their enantioselective addition to ketones has remained a crucial challenge for many decades. As a JSP fellow in 2012, she presented the formation of chiral tertiary alcohols from ketones using a Grignard reagent, a copper(i) catalyst, and a chiral ligand. Using additional activation from a Lewis acid, it is now possible to extend this methodology to the enantioselective 1,4-addition to a wide variety of conjugated electrophiles (unsaturated esters, acids, amides…). In particular, the dearomative addition to *p*-methoxypyridinium ions is a very elegant method to prepare dihydropyridone derivatives.^7^

The comprehensive study of catalytic transformation mechanisms motivates the research group of Prof. Clark Landis (University of Wisconsin–Madison). He introduced his current research by demonstrating the usefulness of the hydroformylation reaction to construct tertiary and quaternary centers in an asymmetric fashion. He developed a new class of rhodium(i) ligands, bisdiazophospholanes, to reach high enantioselectivity and high regioselectivity towards the valuable branched products. While hydroformylation is now a widespread reaction in industry, its complex mechanistic details are not fully understood. Thanks to a unique NMR technique (Wisconsin High-Pressure NMR Reactor or WiHP-NMRR) developed at his university, Prof. Landis proposed a comprehensive microkinetic model for this important transformation.^8^

**Fig. 2:**
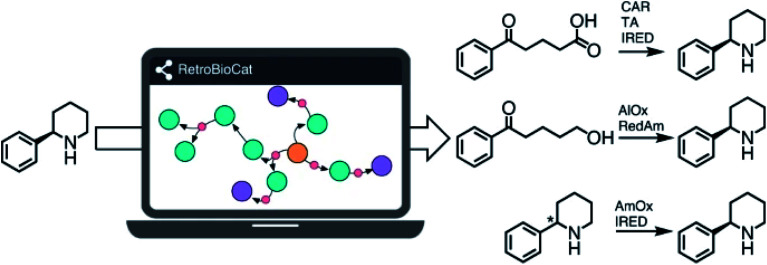
RetroBioCat: a new tool for synthesis planning developed by Prof. Turner. Reproduced from ref. 9 with permission from Springer Nature, © 2021.

We all know that nature is a highly skilled organic chemist, capable of efficient transformations with unparalleled selectivities. Prof. Nicholas Turner (University of Manchester) successfully harnessed this potential to set up a complete biocatalytic toolbox he uses for retrosynthesis designs towards complex molecules (Fig. 2). He convinced us that biocatalysis can work on the industrial scale, as highlighted with his fruitful collaboration with GlaxoSmithKline and Pfizer.^9^

**Fig. 3:**
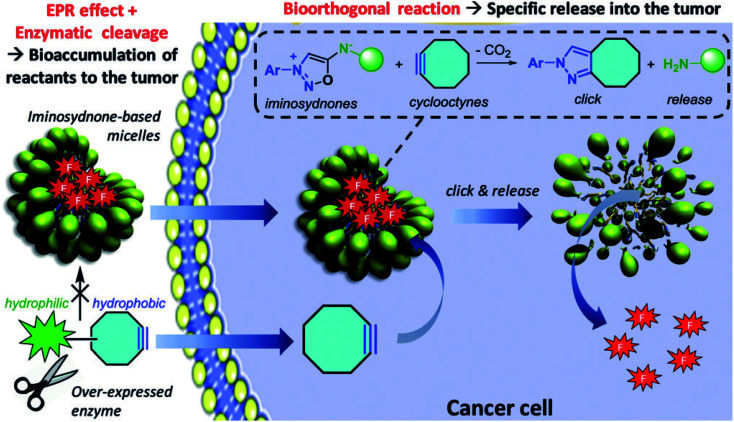
A click and release strategy can bring a micelle payload directly into a tumor. Reproduced from ref. 10 with permission from John Wiley & Sons, Inc., © 2019.

Prof. Frederic Taran (CEA Saclay) presented his impressive research program on the reactivity of mesoionic compounds. He is mainly interested in sydnones, remarkable reagents for click chemistry with alkynes. These reactive compounds readily furnish functionalized pyrazoles by (3 + 2) cycloaddition followed by extrusion of carbon dioxide. He then took advantage of the impressive kinetics of these transformations to develop biorthogonal systems for chemical biology, such as fluorescent probes, radiolabelled compounds and antibodies. He also successfully formed cleavable nanoparticles equipped with an iminosydnone moiety, which can selectively release their content in living systems, at the vicinity of a tumor site (Fig. 3).^10^

Prof. José-Luis Mascareñas (University of Santiago de Compostela), an authority in transition-metal catalysis (Au, Rh, Ru, Pd…), disclosed diverse innovative cycloaddition pathways towards relevant polyclic systems. He built on his expertise to develop a new chemical biology research program and export metal catalysis from organic solvents to biological media, a much more complex and challenging environment. In this context, ruthenium catalysis has been particularly successful to perform redox isomerizations or (2 + 2 + 2) cycloadditions. Prof. Mascareñas presented a gold-catalyzed hydroarylation performed in HeLa cells, which displays an impressive orthogonality to ruthenium catalysis.^11^

## Function

New reactions and molecular insights into chemical processes offer the opportunity to generate complex targets with desired functions. This idea guided the academic career of Prof. Taran and Prof. Mascareñas. They both started their scientific journey by exploring chemical reactivity and then translated this knowledge to the neighboring field of chemical biology. With their experience in synthetic chemistry, they are currently exploring important applications of their previous discoveries to access molecules with biological functions.

Dr Martin Eastgate (Bristol Myers Squibb), the first industrial speaker of the Bürgenstock Conference, follows a similar approach. In the last 30 years, the molecular complexity of industrial targets has significantly increased; thus, new industrial processes have to be developed.^12^ The combination of academic thinking and industrial processes was key to achieve challenging goals, including the replacement of Pd with Ni catalysts or the synthesis of complex chiral pharmaceuticals. An impressive example was the production of new chiral oligonucleotides on an industrial scale, unlocking new phosphorus(v) chemistry.^13^

**Fig. 4:**
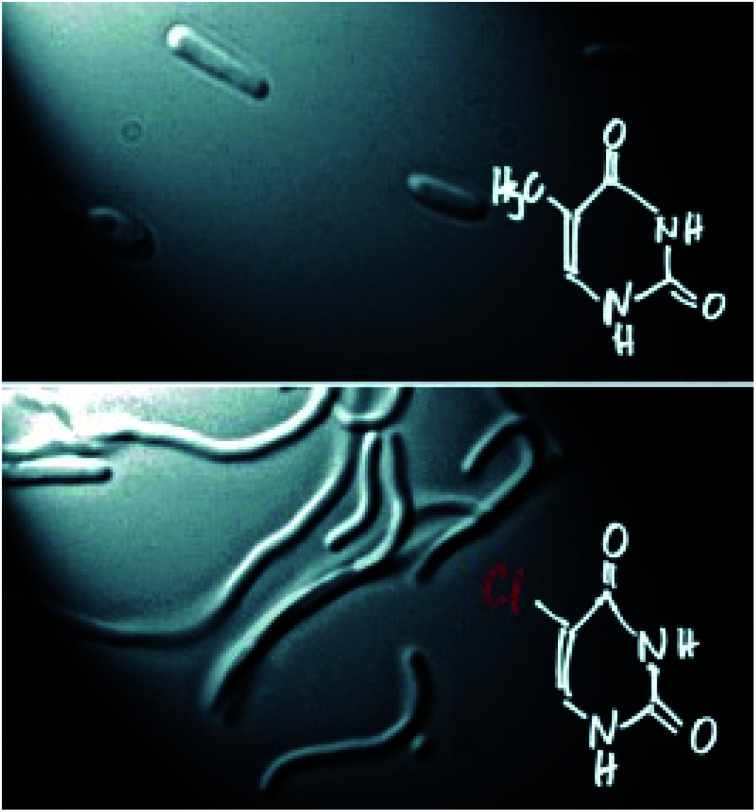
A DNA-modified *E. coli*. Reproduced from ref. 14 with permission from John Wiley & Sons, Inc., © 2011.

The synthesis of modified nucleic acids could be the first step toward the development of new biological systems. This idea was summarized by the visionary lecture given by Prof. Philippe Marliere (TESSI) focused on xenobiology, the study of synthesizing and manipulating biological systems. Inspired by the Jurassic Park quote “life finds a way”, he explained how reprogramming the chemistry of organisms could be possible with ribomorfs (*i.e.* new synthetic base pairs). As a first step in this direction, Prof. Marliere showed a chemically modified *E. coli* whose DNA genome was composed of adenine, cytosine, guanine, and an artificial base, the thymine analogue 5-chlorouracil (Fig. 4).^14^

New reactions are not only important in biology, but also in materials science. With the rising interest in renewable sources, energy storage has become a crucial issue for our society. Prof. Jean-Marie Tarascon (Collège de France) provided an overview of the current knowledge in the field of batteries. Li-ion batteries are at the core of the current technologic revolution and Prof. Tarascon shed light on the mechanism of the anionic redox process responsible for their unparalleled capacity.^15^ Na-ion batteries are also the focus of many research programs, in academia as well as in industry, for their ability to complement the Li-ion technology, offering a useful alternative in a potential Li shortage. The lecture was entitled “Organics in Li-ion batteries: an open call for help”: Prof. Tarascon believes that organic chemists will play a major role in developing new organic electrodes, which currently face numerous challenges (weak electron conductivity, low energy density…). His research group is now developing smart and self-healing batteries with integrated IR sensors capable of detecting a potential material failure.

## Final remarks

The Bürgenstock conference was also an occasion for the participants, including all the JSP fellows, to share their research. Fifty-five posters were presented during two afternoon sessions. As an “appetizer” to each of these sessions, the program included five short presentations from selected speakers. During the Monday session, we had a peek at the research of Paweł Dydio (ISIS, University of Strasbourg), Francesca Paradisi (University of Bern), Matthieu Jouffroy (Johnson & Johnson), Stephan Hacker (Leiden University) and Victor Mougel (ETH Zurich). During the Wednesday session, we discovered some recent results from Rene Koenigs (RWTH Aachen University), Bastien Nay (Ecole Polytechnique), Helena Lundberg (KTH Stockholm), Gabriel Schäfer (Idorsia Pharmaceuticals) and Alastair Lennox (University of Bristol). These appetizer sessions were a great opportunity for the speakers to promote their poster, but they had to be careful, time was ticking: the organizing committee was in charge of starting the short presentation on time and the speakers were gently reminded to wrap up their presentation by the delightful sound of a moo box!

At the end of the conference, Prof. Alois Fürstner was announced as the president for the 56th edition of the Bürgenstock conference in 2023. It was also disclosed that Prof. Erick Carreira (ETH Zurich) will be the vice-president for this next edition and thus the president in 2024.

The long-awaited 55th edition of the conference did not disappoint. Everyone enjoyed finally meeting in-person and discussing chemistry. We would like to thank the speakers for sharing their motivation and recent results in their respective fields. The active participation of all the attendees to the discussion and poster sessions made this conference a truly unique meeting for the chemistry community. We would like to thank the President as well as the organizing committee for putting on such a wonderful event.

The authors are thankful for the JSP fellowships to participate in this event and hope this report will encourage their colleagues to visit wonderful central Switzerland next spring.
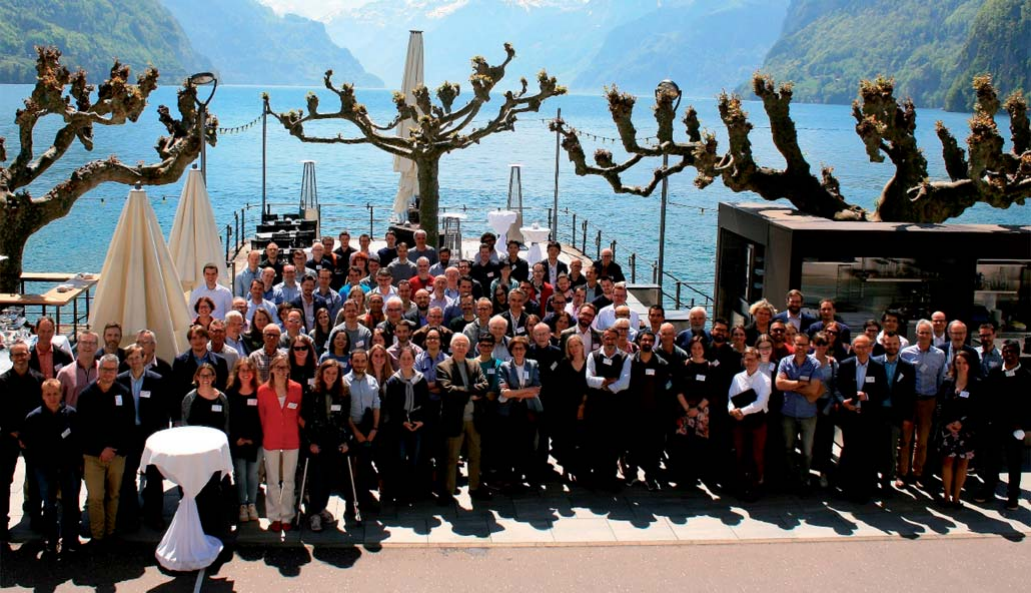


## Supplementary Material
